# Early failure of noninvasive ventilation in chronic obstructive pulmonary disease with acute hypercapnic respiratory failure

**DOI:** 10.1186/2197-425X-3-S1-A671

**Published:** 2015-10-01

**Authors:** H Cho, S Ahn, K Lim, W Kim, Y Lee, B Ko

**Affiliations:** Asan Medical Center, Department of Emergency Medicine, Seoul, Korea, Republic of

## Objectives

Noninvasive ventilation (NIV) in the management of chronic obstructive pulmonary disease (COPD) patient with acute hypercapnic respiratory failure is considered a first-line therapy. However, higher mortality was shown in patients receiving transition from NIV to invasive mechanical ventilation than in patients receiving invasive mechanical ventilation from the first management. We tried to find parameters associated with early NIV failure in patients presenting to ED with acute exacerbation of COPD.

## Methods

Medical records of 218 patients with acute exacerbation of COPD visiting Asan Medical Center and managed with NIV during their stay in the ED from January 2007 to December 2013 were analyzed.

## Results

NIV was successful in 191 (87.6%) and 27 (12.4%) failed NIV treatment. Of the variables obtained before NIV treatment, heart rate (≥120/min: OR 2.8, 95% CI 1.1 - 6.8) and pH (7.25 - 7.29: OR 2.6, 95% CI 1.8 - 8.1; < 7.25: OR 15.2, 95% CI 5.0 - 46.1) were significant factors associated with early NIV failure. Of the variables obtained after 1 hr of NIV treatment, heart rate (≥120/min: OR 4.0, 95% CI 1.4 - 11.5) and pH (7.25 - 7.29: OR 4.7, 95% CI 1.5 - 15.1; < 7.25: OR 29.8, 95% CI 15.7 - 62.7) were still significant.

## Conclusions

Presence of tachycardia and severe acidosis before NIV treatment, and persistence of tachycardia and severe acidosis after 1hour of NIV treatment predicted early NIV failure.
